# Infrared Assisted Production of 3,4-Dihydro-2(1*H*)-pyridones in Solvent-Free Conditions

**DOI:** 10.3390/ijms12042641

**Published:** 2011-04-18

**Authors:** M. Olivia Noguez, Vanessa Marcelino, Hortensia Rodríguez, Osnieski Martín, Joel O. Martínez, Gabriel A. Arroyo, Francisco J. Pérez, Margarita Suárez, René Miranda

**Affiliations:** 1 Departamento de Ciencias Químicas, Facultad de Estudios Superiores Cuautitlán-UNAM, Estado de México, 54754, Mexico; E-Mails: olinoco@yahoo.com.mx (M.O.N.); atlanta126@hotmail.com (J.O.M.); garroyo@unam.mx (G.A.A.); 2 Laboratorio de Síntesis Orgánica, Facultad de Química, Universidad de la Habana, 10400 Ciudad Habana, Cuba; E-Mails: horten@fq.uh.cu (H.R.); osniesky@gmail.com (O.M.); msuarez@fq.uh.cu (M.S.); 3 Instituto de Química, Universidad Nacional Autónoma de México, Ciudad Universitaria, Coyoacán, D.F., 04510, Mexico; E-Mail: japeflo10@hotmail.com

**Keywords:** green approach, infrared irradiation, dihydropyridones, multicomponent reaction, Meldrum’s acid

## Abstract

A green approach for the synthesis of a set of ten 4-aryl substituted-5-alcoxy carbonyl-6-methyl-3,4-dihydro-2(1*H*)-pyridones using Meldrum’s acid has been devised, the absence of solvent and the activation with infrared irradiation in addition to a multicomponent protocol are the main reaction conditions. The transformations proceeded with moderated yields (50–75%) with a reasonable reaction rate (3 h). It is worth noting that two novel molecules of the new class of the *bis*-3,4-dihydropyridones were also obtained. In addition, a comparison without the use of infrared irradiation was performed.

## 1. Introduction

One of the main objectives of green chemistry [1] is to carry out reactions using conditions which are not detrimental to the environment [2]. Following this protocol, an ideal synthesis is one in which a target molecule is produced quantitatively in one step, from readily available and inexpensive starting compounds, in an environmentally acceptable process [3]; it is important to note that multicomponent reactions must be considered as appropriate green methods [4].

On the other hand, Meldrum’s acid or 2,2-dimethyl-1,3-dioxane-4,6-dione (**1**), has attracted considerable attention due to its high acidity and rigid cyclic structure [5]. This versatile molecule is an important substrate in many interesting organic transformations in particular for the production of 4-aryl substituted-5-alcoxycarbonyl-6-methyl-3,4-dihydro-2(1*H*)-pyridones (DHPDOs). This class of compounds containing the 2(1*H*)-pyridone moiety are found in nature, and in some cases, useful biological properties for them have been detected [6]. Consequently, the synthesis of DHPDOs is an area of current interest due to the large number of biologically active molecules of this type [7–9]. In particular, two molecules of commercial interest are Amrinone [10] and Milrinone [11–12]. In addition, several natural compounds with this structure have emerged during the last ten years with potent antitumor [13–15], antifungal [16], antiviral [17] and psychotherapeutic effects [18], along with a new antibiotic [19]. The DHPDOs are also key intermediates in the synthesis of the corresponding pyridines [20]. The title molecules have been prepared by numerous methods [21], e.g., oxidation of *N*-substituted pyridinium salts [22], by means of Knoevenagel-type reactions [23] such as the cross-condensation of cyanoacetoamide and β-dicarbonyl compounds with basic catalysts or by the reaction of 2-pyrones with amides. Despite the large number of known methods for their synthesis, the importance of this class of molecules means that new procedures must always be welcomed, in particular using suitable green techniques [24–26], in order to be less aggressive to the environment.

**Scheme 1.**
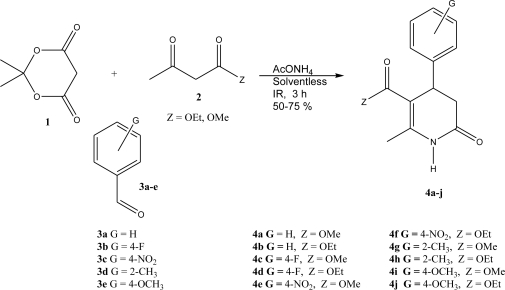


**Scheme 2.**
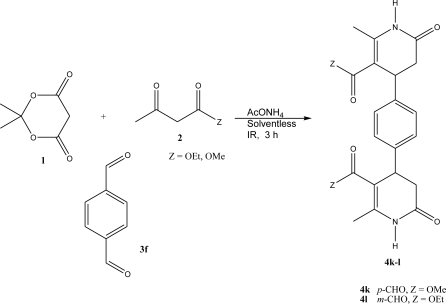


As a part of our research program, in particular by the employment of infrared irradiation as the activating mode of the reaction, we have been interested in the production of heterocyclic compounds with important pharmacological effects [27–30], mainly using eco-conditions. Thus, the goal of this paper is to present a new mode for the synthesis of a set of ten 3,4-dihidro-2(1*H*)-pyridones (**4a–j**), in addition to the two novel *bis*-3,4-dihydro-2(1*H*)-pyridones (**4k–l**), using solvent-free conditions and for the first time the activation with infrared irradiation, for this purpose, as summarized in Schemes 1–2. This methodology must be considered as a green approach for the production of these classes of molecules, validating the employment of infrared irradiation as a nonclassical heating technique for many organic transformations.

## 2. Results and Discussion

The results of this new and ecologically sound method for the production of this series of twelve 4-aryl substituted-5-alcoxycarbonyl-6-methyl-3,4-dihydro-2(1*H*)-pyridones, are summarized in Table 1.

As it can be seen, a wide range of aldehydes undergo this reaction with moderate yields (50–75%) of the corresponding products. The known molecules (**4a–j**) (Scheme 1) were identified by correlating the corresponding physical and spectroscopic data with literature values [31–36]. Moreover, this procedure must be considered as a new and simple method with an appropriately green approach, since no solvent was employed, in addition to the use of infrared irradiation to give a clean and rapid activation of this multicomponent reaction.

In addition, an appropriate comparison with the corresponding thermal reactions was also performed using a heating mantle (neat, same time and temperature); no more than 40% of substrate transformation was achieved, maybe because heat transference by the thermal method is less effective.

The molecules were structurally confirmed by NMR and MS studies. The ^1^H-NMR spectra of these compounds show the two proton on C-3 confirmed by a doublet of doublets at δ 4.4–4.7 corresponding to the proton on C-4 subject to the splitting by coupling with the protons C-3a,b. It is important to note that, in general, the corresponding signal of the proton C-4 appears as a doublet due to weak coupling with one of the protons C-3a, b (*J*_3b,4_ = 1.9 Hz and *J*_3a,4_ = 8.3 Hz). In this sense, the signals of the protons C-3 correspond to a doublet of doublets between δ 2.8–3.2. The ^13^C-NMR spectra of the title compounds show the signals of the olefinic carbons at 104 and 148 assigned to C-5 and C-6, respectively. Other characteristic signals were observed at δ ≈ 169 and δ ≈ 166 corresponding to C-2 and C-8, respectively [35].

Along with the assignment of the MS data of the compounds studied, the corresponding molecular ions M^+^ were observed with significant relative abundances (25–83). In addition, a series of common fragments were observed, including [M-CH_3_]^+^, [M-OCH_3_]^+^ and [M-OCH_2_CH_3_]^+^. Moreover, it is important to note that structures of the new *bis*-3,4-dihydropyridones (**4k–l**) (Scheme 2) were also confirmed by their high resolution mass spectrometry data.

## 3. Experimental Section

The aldehydes, used as substrates, are commercially available (Sigma-Aldrich Chemical Co.) and were employed without further purification. The reactions were monitored by *tlc* on percolated (0.25 mm) Merck silica-gel 60-F_254_ aluminum sheets. In general, the product visualization was done using a 254 and 365 nm UV lamp, I_2_ or CeSO_4_·H_2_SO_4_ 1%. Melting points, uncorrected, were measured using a Fisher Scientific apparatus. The mass spectrometric analyses (EIMS, HRMS) were performed using a JEOL MStation JMS-700 mass spectrometer with a source temperature of 230 °C, an ionization energy of 70 eV and an ionization trap current of 300 μA. In the HRMS studies, perfluorokerosene was used as internal mass reference. The corresponding range of mass measurements was set so as to include the two standard peaks that encompassed the sample peak of interest, in addition the mass resolution and scan speed used were 30,000 (10% valley) and 60 s/decade, respectively. The accurate mass was calculated as the average of the values measured in 5–10 scans, determined from the mass centroids of the M^+•^ ion and the other peaks. The corresponding elemental composition data was calculated within a mass window of ±10 ppm from the measured accurate mass using the program installed in the data system. Thus the elemental composition with a mass that best fitted the measured value and that made chemical sense was assigned to the ion of interest. NMR experiments were carried out using a Varian Mercury-300 at 300 MHz and 75 MHz for hydrogen and carbon respectively, the solvents used were DMSO-d6 and CDCl_3_; TMS was employed as the internal reference. The infrared irradiation was performed using a Phillips IR lamp (375 W/220 V) integrated to an infrared reactor [27–28], designed by our research group [29–30] and validated by a wide number of applications summarized in a recent review [31].

**General Method: (Table 1, entry 4a–j)** A mixture of Meldrum’s acid, methyl or ethyl acetoacetate, substituted benzaldehyde and ammonium acetate (1 mmol of each reagent), were thoroughly mixed in a round-bottomed flask (50 mL), equipped with a condenser. This mixture was exposed to infrared irradiation for 3 h. The reactions were monitored by *tlc* using *n*-hexane: ethyl acetate (80:20 or 60:40) as eluent. The purification of the products was carried out by means of preparative chromatography, using *n*-hexane/ethyl acetate (80:20 or 60:40) as eluent; the product was dissolved in acetone and then supported on percolated Merck silica-gel 60-F_230_ glass sheets. For the production of **4k–l** were employed 2 mmol of Meldrum’s acid, methyl or ethyl acetoacetate, ammonium acetate and 1 mmol of the *para*-dialdehyde.

### 4-(4’-Fluorophenyl)-5-methoxycarbonyl-6-methyl-3,4-dihydro-2(1*H*)-pyridone (4c)

**¹H-NMR** (DMSO-*d*6/TMS, δ ppm): 9.95 (s, 1H, NH); 7.31–7.06 (m, 4H, Ar); 4.72 (d, 1H, H-4, *J* = 8.1 Hz); 4.20 (s, 3H, OCH_3_-9); 2.93 (dd, 1H, H-3a, *J* = 16.3 Hz, *J* = 8.1 Hz), 2.69 (d, 1H, H-3b); 2.33 (s, 3H, CH_3_-7).

**^13^C-NMR** (CDCl_3_-*d*6/TMS, δ ppm): 169.7 (C-2); 166.9 (C=O); 164.8 (C-4’); 148.8 (C-6); 138.5; 131.8; 128.4 (C-Ar); 106.6 (C-5); 52.1 (CH_3_-9); 38.3 (C-3); 36.6 (C-4); 18.9 (C-7).

**EI-MS m/z** (relative abundance %): ^·^263(25) M^+^; 204(100); 149(80) [M-114]^+^; 122(50) [M-141]^+^.

### 5-Ethoxycarbonyl 4-(4’-fluorophenyl)-6-methyl-3,4-dihydro-2(1*H*)-pyridone (4d)

**¹H-NMR** (DMSO-*d*6/TMS, δ ppm): 9.93 (s, 1H, NH); 7.21–7.06 (m, 4H, Ar); 4.12 (dd, 1H, H-4, *J* = 7.4 Hz); 4.01 (q, 2H, CH_2_-9); 2.93(dd, 1H, H-3a, *J* = 16.0 Hz, *J* = 7.4 Hz), 2.70 (dd, 1H, H-3b, *J* = 16.0 Hz); 2.31 (s, 3H, CH_3_-7); 1.09 (t, 3H, CH_3_-10).

**^13^C-NMR** (DMSO-*d*6/TMS, δ ppm): 169.6 (C-2); 166.4 (C-8); 148.3 (C-6); 138.9 (C-4’); 128.4; 115.4; 115.0 (C-Ar); 104.9 (C-5); 59.4 (CH_3_-9); 38.2 (C-3), 36.7 (C-4); 18.3 (C-7); 14.1 (C-10).

**EI-MS m/z** (relative abundance %): 277(75) M^+^; 248(85)[M-29]^+^; 231(55) [M-46]^+^; 204(100).

### 6-Methyl-4-(2’-methylphenyl)-5-methoxycarbonyl-3,4-dihydro-2(1*H*)-pyridone (4g)

**¹H-NMR** (DMSO-*d*6/TMS, δ ppm): 9.95 (s, 1H, NH); 7.18–6.88 (m, 4H, Ar); 4.31 (d, 1H, H-4, *J* = 8.2 Hz); 3.49 (s, 3H, CH_3_-9); 2.95 (dd, 1H, H-3a, *J* = 16.3Hz; *J* = 8.2 Hz); 2.45 (d, 1H, H-3b, *J* = 16.3 Hz); 2.36(s, 3H, CH_3_-2’); 2.34 (s, 3H, CH_3_-7).

**^13^C-NMR** (DMSO*d*6, δ ppm): 169.5 (C-2); 169.9 (C-8); 148.9 (C-6); 140.4; 134.9; 130.8; 126.6; 126.2; 125.1 (C-Ar); 104.8 (C-5); 51.1 (CH_3_-9); 36.9 (C-3); 33.9 (C-4); 18.9(CH_3_-C-2’); 18.1 (C-7).

**EI-MS m/z** (relative abundance %): 259(55) M^+^; 244(10) [M-15]^+^; 200(65); 115(40) [M-114]^+^; 43(100) [M-216]^+^.

### 5-Ethoxycarbonyl-6-methyl-4-(2’-methylphenyl)-3,4-dihydro-2(1*H*)-pyridone (4h)

**¹H-NMR** (DMSO-*d*6/TMS, δ ppm): 9.94 (s, 1H, NH); 7.20–6.89 (m, 4H, Ar); 4.32 (dd, 1H, H-4, *J* = 8.2 Hz); 3.93 (q, 3H, OCH_2_-9); 2.94 (dd, 1H, H-3a, *J* = 16.4 Hz, *J* = 8.2 Hz), 2.40 (dd, 1H, H-3b, *J* = 16.4 Hz); 2.36 (s,3H, CH_3_-C2’); 2.34 (s, 3H, CH_3_-7); 1.39 (t, 3H, CH_3_-10).

**^13^C-NMR**. (DMSO-*d*6/TMS, δ ppm): 169.4 (C-2); 166.4 (C-8); 148.5 (C-6); 140.7; 134.8; 130.7; 126.5; 126.2; 125.1 (C-Ar); 105.1 (C-5); 59.4 (CH_2_-9); 38.2 (C-3); 33.8 (C-4); 18.9 (CH_3_-C2’); 18.2 (C-7); 14.0 (C-10).

**EI-MS m/z** (relative abundance %): 273(80) M^+^; 258(10) [M-15]^+^; 244(50) [M-29]^+^; 212(80) [M-61]^+^; 200(100).

### Phenylene-1’,4’-di-(4-(6-methyl-5-methoxycarbonyl-3,4-dihydro-2(1*H*)-pyridone)) (4k)

**¹H-NMR** (DMSO-*d*6/TMS, δ ppm): 9.97 (s, 1H, NH); 7.41–6.93 (m, 4H, Ar); 4.17–3.94 (m, 6H, H-4, OCH_2_-9); 2.51 (dd, 1H, H-3a); 2.21 (s, 3H, CH_3_-7); 1.07 (t, 3H, CH_3_-10).

**^13^C-NMR**. (DMSO-*d*6/TMS, δ ppm): 169.6 (C-2); 163.7 (C-8); 148.0 (C-6); 140.9; 134.8; 128.2; 127.0; 126.7; (C-Ar); 104.9 (C-5); 59.3 (CH_2_-9); 38.6 (C-3); 37.3 (C-4); 18.9 (CH_3_-C2’); 18.2 (C-7); 14.1 (C-10).

**EI-MS m/z** (relative abundance %): 412(0) M^+^; 381(5) [M-31]^+^; 354(7) [M-61]^+^; 245(20) [C_14_H_15_O_3_N]^+^; 169(15)[C_8_H_10_O_2_N]^+^.

**HR-MS:** observed 412.1628, expected 412.1634, C_22_H_24_N_2_O_6_.

### Phenylene-1’,4’-di-(4-(5-ethoxycarbonyl-6-methyl-3,4-dihydro-2(1*H*)-pyridone)) (4l)

**¹H-NMR** (DMSO-*d*6/TMS, δ ppm): 9.84 (s, 1H, NH); 7.40–6.90 (m, 4H, Ar); 4.20–3.90 (m, 6H, H-4, OCH_2_-9); 2.94 (dd, 1H, H-3a); 2.25 (s, 3H, CH_3_-7); 1.05 (t, 3H, CH_3_-10).

**^13^C-NMR**. (DMSO-*d*6/TMS, δ ppm): 169.7 (C-2); 167.5 (C-8); 148.0 (C-6); 140.9; 134.8; 128.3; 127.0; 126.7; (C-Ar); 104.9 (C-5); 59.4 (CH_2_-9); 38.3 (C-3); 36.9 (C-4); 18.9 (CH_3_-C2’); 18.2 (C-7); 14.1 (C-10).

**EI-MS m/z** (relative abundance %): 440(5) M^+^; 396(4) [M-OEt]^+^; 367(5) [M-CO_2_Et]^+^; 259(10) [M-C_9_H_12_O_2_N]^+^; 182(5)[C_9_H_12_O_2_N]^+^.

**HR-MS:** observed 440.1941, expected 440.1947, C_24_H_28_N_2_O_6_.

## 4. Conclusions

This procedure for the production of 3,4-dihydro-2(1*H*)-pyridones should be regarded as a new and simple method with an appropriately green approach, since no solvent was employed. This, in addition to the use of infrared irradiation, provides a clean and rapid mode for the activation of this multicomponent reaction. Finally, this work is another contribution detailing the employment of infrared irradiation as a nonclassical heating technique for the transformation of many organic molecules.

## Figures and Tables

**Table 1.** Dihydropyridones (**4a–j**) produced under infrared irradiation^a^.

**Compound**	**Melting point^[b]^ (°C)**	**Yield^[c]^%**
**4a**	197–198(197–198) [12,28]	75
**4b**	168–171	75
**4c**	206–208(207–209) [12,28]	75
**4d**	184–186(184–186) [29]	70
**4e**	200–202(201–201) [29]	75
**4f**	130–133	60
**4g**	oil	70
**4h**	oil	60
**4i**	180–183	55
**4j**	185–188	50
**4k**	oil	65
**4l**	oil	59

[a] 3 hours

[b] Experimental

[c] Isolated pure product.
